# Evidence for transgenerational metabolic programming in *Drosophila*

**DOI:** 10.1242/dmm.011924

**Published:** 2013-05-02

**Authors:** Jessica L. Buescher, Laura P. Musselman, Christina A. Wilson, Tieming Lang, Madeline Keleher, Thomas J. Baranski, Jennifer G. Duncan

**Affiliations:** 1 Department of Pediatrics, and; 2 Department of Internal Medicine, Washington University School of Medicine, St Louis, MO 63110, USA

## Abstract

Worldwide epidemiologic studies have repeatedly demonstrated an association between prenatal nutritional environment, birth weight and susceptibility to adult diseases including obesity, cardiovascular disease and type 2 diabetes. Despite advances in mammalian model systems, the molecular mechanisms underlying this phenomenon are unclear, but might involve programming mechanisms such as epigenetics. Here we describe a new system for evaluating metabolic programming mechanisms using a simple, genetically tractable *Drosophila* model. We examined the effect of maternal caloric excess on offspring and found that a high-sugar maternal diet alters body composition of larval offspring for at least two generations, augments an obese-like phenotype under suboptimal (high-calorie) feeding conditions in adult offspring, and modifies expression of metabolic genes. Our data indicate that nutritional programming mechanisms could be highly conserved and support the use of *Drosophila* as a model for evaluating the underlying genetic and epigenetic contributions to this phenomenon.

## INTRODUCTION

Obesity is a major risk factor for metabolic syndrome and type 2 diabetes, and is increasing at a staggering rate in young populations. The Centers for Disease Control and Prevention reported that obesity prevalence among US children and adolescents has nearly tripled since 1980. Childhood obesity often goes unresolved in adulthood and frequently leads to chronic secondary health problems, including type 2 diabetes and cardiovascular disease (Baker et al., 2007; Field et al., 2005; Juonala et al., 2011). A predisposition to obesity and its comorbidities might be established *in utero*, owing to a suboptimal maternal-fetal nutritional environment (McMillen and Robinson, 2005). Indeed, epidemiological evidence demonstrates a strong relationship between prenatal nutritional environment, birth weight and adult disease susceptibility, with increased risks for adult disease existing at both low and high birth weights and also in cases of gestational diabetes (McMillen and Robinson, 2005; Reece, 2010; Sullivan et al., 2011). It is postulated that, in order to maintain energy homeostasis during nutrient deprivation or nutrient excess, alterations occur in fetal metabolic programming mechanisms, which could become maladaptive, resulting in postnatal pathology (McMillen and Robinson, 2005; Symonds et al., 2009; Warner and Ozanne, 2010). Consequently, therapeutic intervention during early stages of development represents a promising prevention strategy for a number of increasingly prevalent health concerns.

Such epidemiological observations have been corroborated in mammalian models, yet little is known of the molecular mechanisms underlying this phenomenon (Mitchell et al., 2009; Rajia et al., 2010; Simmons et al., 2001; Tamashiro et al., 2009). Genetically tractable model organisms, such as *Drosophila melanogaster*, offer unique opportunities to study the impact of nutrition on metabolism. *Drosophila* possess key metabolic organ systems and share many conserved metabolic functions with vertebrates, including analogous insulin, insulin-like-growth-factor and target of rapamycin (TOR) signaling pathways, and regulation of circulating sugars, energy storage and energy mobilization (Baker and Thummel, 2007; Schlegel and Stainier, 2007). Recent advances from *Drosophila* have provided insight into the complex relationship between nutritional environment, gene expression and metabolism (Birse et al., 2010; Bujold et al., 2010; Fujikawa et al., 2009; Horner et al., 2009; Musselman et al., 2011; Ruaud et al., 2011; Sieber and Thummel, 2009). However, transgenerational effects of nutrition have not been reported. Herein, we describe a novel transgenerational *Drosophila* model in which offspring from maternal flies subjected to caloric excess demonstrate disrupted metabolic homeostasis that is associated with transcriptional changes of metabolic regulators.

## RESULTS

### A high-sugar diet induces obesity in adult female flies

In order to establish a *Drosophila* model for examining transgenerational effects of caloric excess, we first examined the effect of a high-calorie diet on body composition in virgin *w^1118^* female flies. Within 24 hours of eclosion, *w^1118^ Drosophila* virgin female flies were placed on semi-defined food that was supplemented with either 0.15 mol/l sucrose [low sugar (LS) control] or 1 mol/l sucrose [high sugar (HS)] for 7 days and were subsequently examined for changes in body composition. This semi-defined food medium has previously been described (Backhaus et al., 1984) and was originally developed for the purpose of evaluating nutritional and metabolic changes in *Drosophila*, while avoiding the frequent variability found between food batches of stock foods containing molasses and cornmeal. Thus, the diets we chose use this semi-defined food as the base with greater than sixfold more sucrose in the HS diet. An HS diet has been shown to elicit obesity and insulin resistance in *Drosophila* larvae (Musselman et al., 2011; Pasco and Léopold, 2012). Additionally, adult flies cultured on HS food for 3 weeks were recently reported to develop metabolic defects and cardiomyopathy (Na et al., 2013). However, the effects of an HS diet specifically on reproductive maternal flies were not examined.

**TRANSLATIONAL IMPACT**

**Clinical issue**

Several epidemiologic studies have linked a suboptimal prenatal nutritional environment with susceptibility to metabolic and cardiovascular disease in adulthood. This concept originated with David Barker’s work demonstrating the impact of maternal malnutrition on metabolic disease risk in offspring, but it is now apparent that maternal obesity and diabetes also predispose to childhood obesity and development of diabetes. Unfortunately, maternal obesity is now extraordinarily common, and rates of obesity are increasing at alarming rates in children. It has been postulated that a suboptimal prenatal environment can drive metabolic reprogramming events that make offspring more susceptible to obesity and metabolic disease (e.g. type 2 diabetes). Understanding the mechanisms that underlie this type of programming will allow us to therapeutically intervene at earlier stages to prevent these serious diseases.

**Results**

To understand the basic mechanisms underlying nutritional programming, the authors established a new transgenerational *Drosophila* model that facilitates detailed examination of the pathways involved in altered metabolism. Using this model, they show that a maternal diet that is high in sugar results in increased carbohydrate storage as well as decreased cholesterol storage in developing offspring. Similarly, adult offspring were found to accumulate increased triglyceride levels when challenged with a high-sugar diet. In addition, the authors demonstrate that expression of many genes involved in metabolism is altered in offspring whose mothers were fed a high-sugar diet. Finally, the authors observed similar changes in second-generation offspring, suggesting that obesity can be inherited through multiple generations.

**Implications and future directions**

These results strongly support the idea that maternal diet affects metabolism in offspring. The offspring of *Drosophila* that are fed a high-sugar diet are predisposed to accumulating excess fat, indicating that, like humans, they are prone to obesity. The altered expression of metabolic genes provides clues to some of the pathways involved in mediating these changes, thereby paving the way for further investigation into potential candidates that could be exploited to develop new therapeutic strategies. The establishment of a simple and experimentally tractable fly model will enable rapid elucidation of the mechanisms and pathways involved in metabolic programming, which could then be readily translated to mammalian systems.

**Fig. 1.**
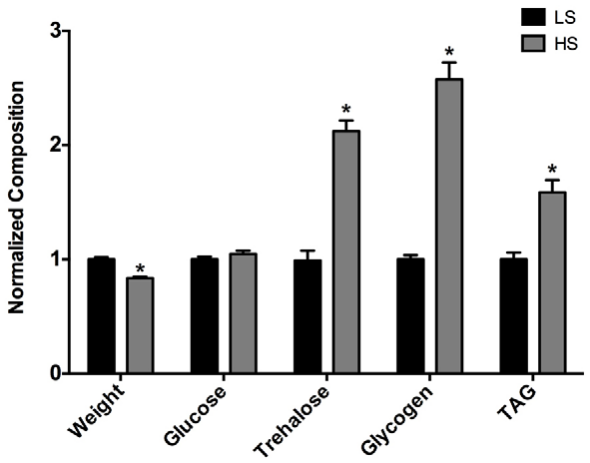
**HS-fed virgin females exhibit an obese-like phenotype.** Virgin female body composition after 7 days of low sucrose (LS) or high sucrose (HS) diet. Bars represent mean body composition, normalized to weight and presented as fold change versus LS control ± s.e.m.; *n*=13–15 pooled samples; *P<0.05.

We found that virgin females subjected to an HS diet for 7 days during adulthood weighed less compared with the LS-fed females (Fig. 1). This decreased weight likely reflects the physiological changes that result in increased morbidity and mortality associated with longer-term HS-diet feeding as previously observed by the Baranski and Cagan labs (data not shown) (Na et al., 2013). Specifically, Na et al. noted that flies that fed for just 3 weeks on an HS diet had increased cardiac arrhythmia and that HS-fed flies overall had a decreased lifespan (Na et al., 2013). Thus, the decreased weight in the virgin females probably reflects early morbidity associated with the diet. Females fed an HS diet for 7 days exhibited increased whole body trehalose, whereas glucose remained unchanged, and an increase in glycogen (Fig. 1). Furthermore, triacylglycerol (TAG) was markedly increased in HS-fed flies (Fig. 1), representing a marked increase in percent body fat. Thus, a 7-day HS diet elicits an obese-like phenotype in female flies.

### Larval offspring from HS-fed maternal flies exhibit altered body composition

**Fig. 2.**
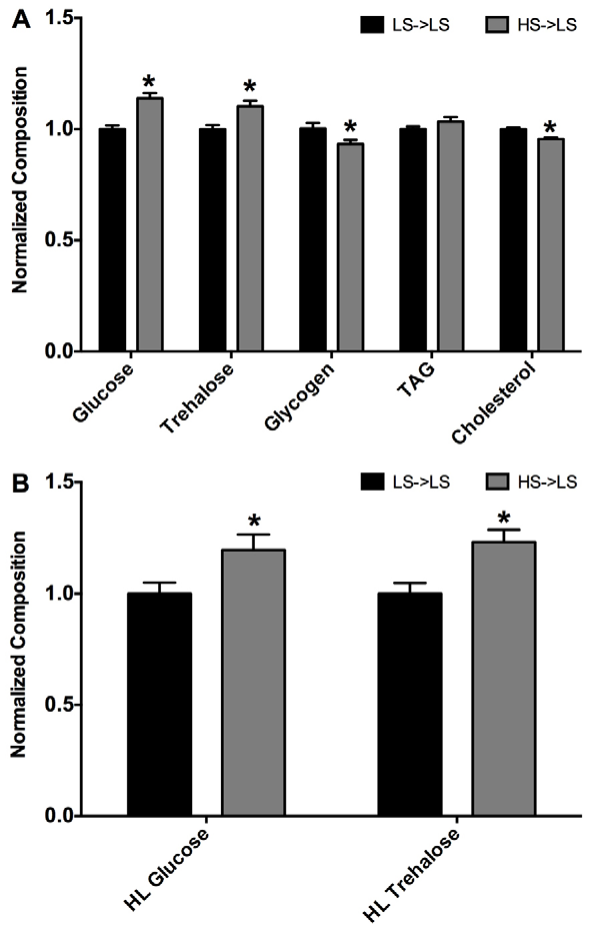
**Male larval offspring from HS-fed maternal flies have altered body composition and circulating sugar levels.** (A) Wandering L3 (wL3) total body composition. Bars represent mean body composition, normalized to weight and presented as fold change relative to LS-LS control ± s.e.m.; *n*≥25. (B) Hemolymph glucose and trehalose concentrations from wL3 offspring. Bars represent mean concentration as compared with controls (LS-LS=1.0) ± s.e.m.; *n*=16–18 pooled samples; * *P*<0.05.

To evaluate the impact of the maternal HS diet on offspring, we next investigated body composition of offspring from LS- and HS-fed maternal flies. Virgin female flies were transferred to LS food after 7 days of control LS food or HS food and mated with *w^1118^* male flies for 24 hours. Importantly, all offspring developed on an LS diet and were never directly exposed to the HS diet, such that any alterations in body composition would be due only to the diet of the maternal flies. We selected wandering L3 (wL3) offspring for body composition studies because these animals have completed development and are no longer feeding. Thus, any contribution of maternal diet to offspring body composition at this stage is more likely to represent persistent changes rather than reflect nutrient or mRNA deposition in the embryo. Examination of total body composition of wL3 offspring found that offspring from HS-fed females (HS-LS offspring) had no change in body weight (data not shown), but exhibited a mild but significant elevation in whole body glucose and trehalose, whereas glycogen was slightly decreased when compared with control offspring from LS-fed females (LS-LS offspring) (Fig. 2A). Additionally, wL3 male HS-LS offspring showed no changes in TAG, but displayed a modest yet significant reduction in cholesterol (Fig. 2A) compared with control LS-LS animals. To examine circulating sugars in offspring, we extracted hemolymph from wL3 males and measured glucose and trehalose content. Both hemolymph glucose and hemolymph trehalose were significantly increased in HS-LS when compared with control LS-LS animals (Fig. 2B). Hence, HS-fed maternal flies generate wL3 male offspring that exhibit elevated glucose and trehalose in both total body and hemolymph. Taken together, these data suggest that HS maternal diet alters carbohydrate homeostasis in developing offspring.

### Adult offspring from HS-fed maternal flies exhibit altered body composition when challenged with an HS diet

**Fig. 3.**
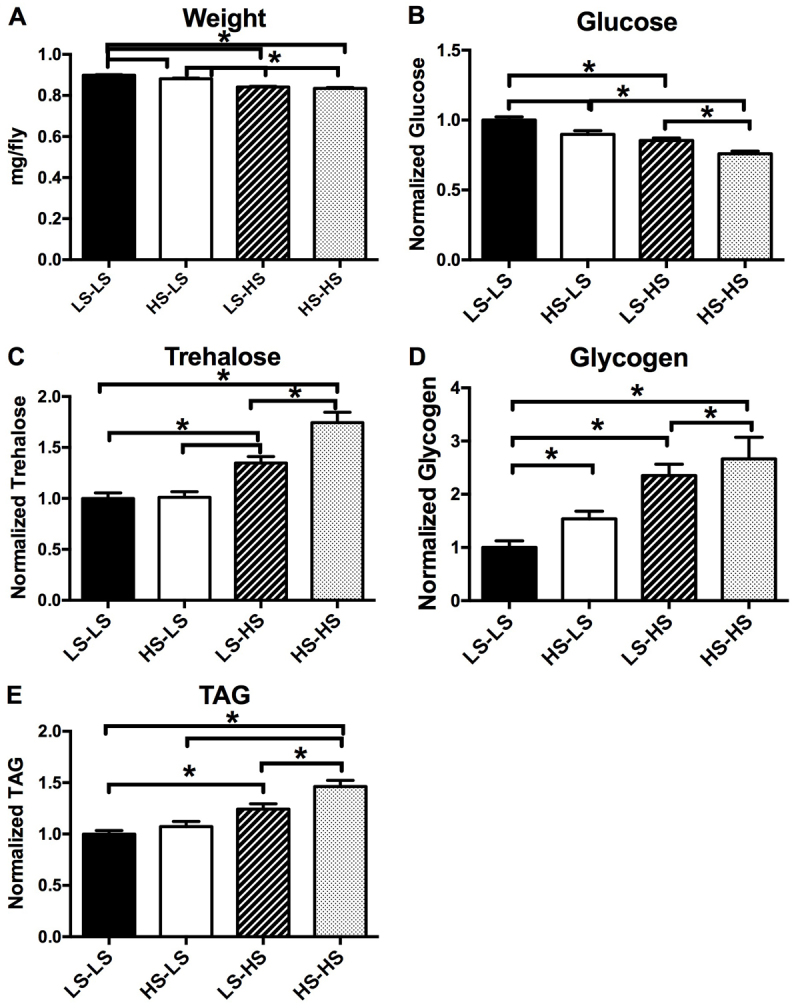
**Diet-challenged offspring from HS-fed maternal flies exhibit altered body composition.** (A–E) Total body composition of adult male offspring after 14 days of diet challenge with either low sucrose (LS) or high sucrose (HS) diet. *x*-axis labels denote maternal diet-offspring adult diet after eclosion. Bars represent mean body composition, normalized to weight and presented as fold change versus control (LS-LS=1.0) ± s.e.m.; *n*≥20 pooled samples; *P*<0.05 versus *LS-LS.

To determine whether metabolic defects persisted into adulthood, we next examined body composition of adult male offspring. Adult male offspring reared on LS food as larvae were cultured on either LS or HS food for 14 days after eclosion and then analyzed. When adult offspring were cultured on LS food, offspring from HS-fed maternal flies (HS-LS) displayed a slight reduction in body weight (Fig. 3A) and glucose (Fig. 3B) and no changes in trehalose or TAG as compared with controls (LS-LS) (Fig. 3C,E). Glycogen, however, was significantly increased under LS culture conditions (Fig. 3D), indicating that defective carbohydrate homeostasis persists into adulthood. To further investigate carbohydrate homeostasis, we challenged offspring with an HS diet and examined the effects on body composition. In offspring from LS-fed maternal flies, an HS diet (LS-HS) elicited a further reduction in weight and glucose (Fig. 3A,B) and an increase in trehalose, glycogen and TAG as compared with LS-LS flies (Fig. 3C–E). When offspring from HS-fed maternal flies were cultured on HS food (HS-HS), changes in body composition were more pronounced. Specifically, an HS diet caused a greater reduction in glucose in HS-HS offspring than that seen in LS-HS (Fig. 3B). This reduction was also much greater than that observed in HS-LS. Additionally, trehalose and TAG were both increased to levels above those observed in LS-HS offspring (Fig. 3C,E). These data suggest that offspring from HS-fed maternal flies are predisposed to developing an obese-like phenotype when exposed to caloric excess. It is notable that the adult fat body, the key nutrient storage tissue in the fly, is not derived from the larval fat body; rather, larval fat body cells undergo cell death during the first few days of adult life (Aguila et al., 2007). Therefore, the changes seen in adult body composition are unlikely to represent macromolecules stored during the larval stage, but rather represent altered programming of nutrient storage.

### Maternal HS diet results in differential gene expression in male offspring

**Fig. 4.**
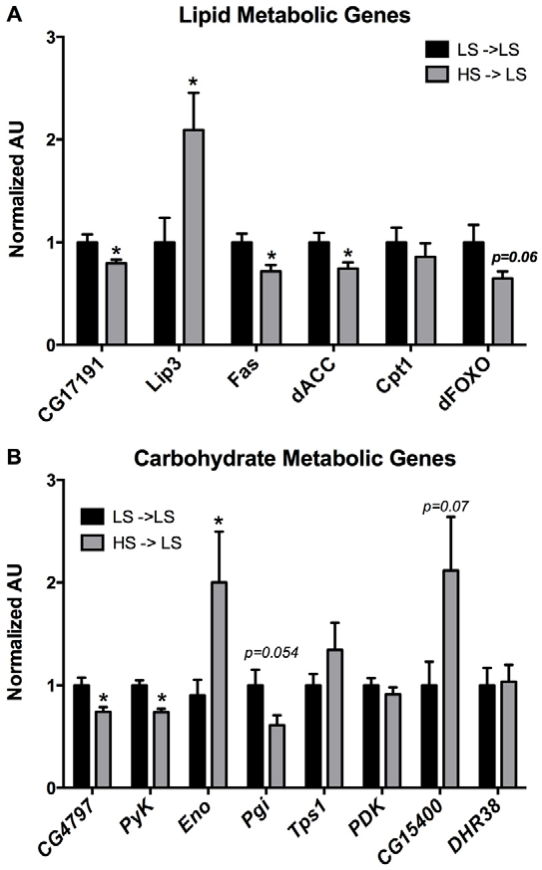
**wL3 male offspring from HS-fed maternal flies exhibit altered gene expression.** (A,B) qRT-PCR analysis of mRNA from mL3 male offspring for genes involved in lipid metabolism (*CG17191*, *Lip3*, *Fas*, *dACC*, *Cpt1*, *dFOXO*) and carbohydrate metabolism [*CG4797* (glucose transporter), *PyK*, *Eno*, *Pgi*, *Tps1*, *Pdk*, *CG15400* (glucose-6-phosphatase), *DHR38*]. Bars represent relative expression ± s.e.m., presented as fold change compared with the control value (=1.0) in each case. All expression data was normalized to α-tubulin 84B mRNA. *n*=8–10 pooled samples; *P<0.05.

To better understand the molecular mechanisms underlying the dynamic alterations in offspring metabolism resulting from an HS maternal diet, we evaluated gene expression by quantitative real-time reverse transcriptase PCR (qRT-PCR). Target genes were selected based on previous microarray data obtained from studies of larval offspring reared on an HS diet (Musselman et al., 2011), and also based on the body composition changes that were seen, which suggested specific metabolic pathways as potentially regulated. We examined genes involved in TAG and carbohydrate metabolism in both mid-third instar (mL3) male larval offspring and adult male diet-challenged offspring. mL3 animals were chosen because this is a state of high metabolic activity and previous gene expression studies with larvae reared on an HS diet had shown that a larger number of genes were differentially regulated at this stage (Musselman et al., 2011). In mL3 animals, which were exposed only to an LS diet, we examined lipases, *CG17191* and *CG8823* (*Lip3*) (Fig. 4A). *CG17191* was downregulated, whereas expression of *Lip3* was markedly increased in offspring from HS-fed maternal flies, compared with controls. We also observed downregulation of genes involved in fatty acid synthesis: *CG3523* [fatty acid synthase (*Fas*)] and *CG11198* [acetyl-CoA carboxylase (*dACC*)]. *dFOXO* (*CG3143*), a forkhead transcription factor implicated in both lipid and carbohydrate metabolism (Jünger et al., 2003; Xu et al., 2012), exhibited mildly reduced expression (*P*=0.06). There was no change in expression of the fatty acid oxidation gene *CG12891* (*CPT1*).

We further examined genes involved in carbohydrate metabolism (Fig. 4B). Expression of a putative sugar transporter, *CG4797*, was decreased in offspring from HS-fed maternal flies. Glycolytic genes also exhibited differential expression when compared with control animals: *CG17654* [enolase (*Eno*)] was upregulated, whereas *CG8251* [phosphoglucose isomerase (*Pgi*)] and *CG7070* [pyruvate kinase (*PyK*)] were downregulated. *Tps1* [trehalose-6-phosphate synthase 1 (*CG4104*)], which is involved in the conversion of glucose to trehalose, was slightly increased but this was not statistically significant. Finally, evaluation of gene expression for gluconeogenesis effector enzymes showed a trend towards upregulation of *CG15400* (glucose-6-phosphatase), which catalyzes the final step in gluconeogenesis and glycogenolysis. Levels of *CG10924* [predicted phosphoenolpyruvate carboxykinase (PEPCK) activity] and *CG5165* [phosphoglucomutase (*PGM-1*)] were not significantly different compared with controls, but both had slightly decreased expression. We also evaluated other lipases (*CG5932*, *brummer*), the *Drosophila* insulin like peptides (dILPs) and Niemann-Pick (NPC) genes. Only *NPC1b* trended towards a decrease in HS larvae (*P*=0.08).

**Fig. 5.**
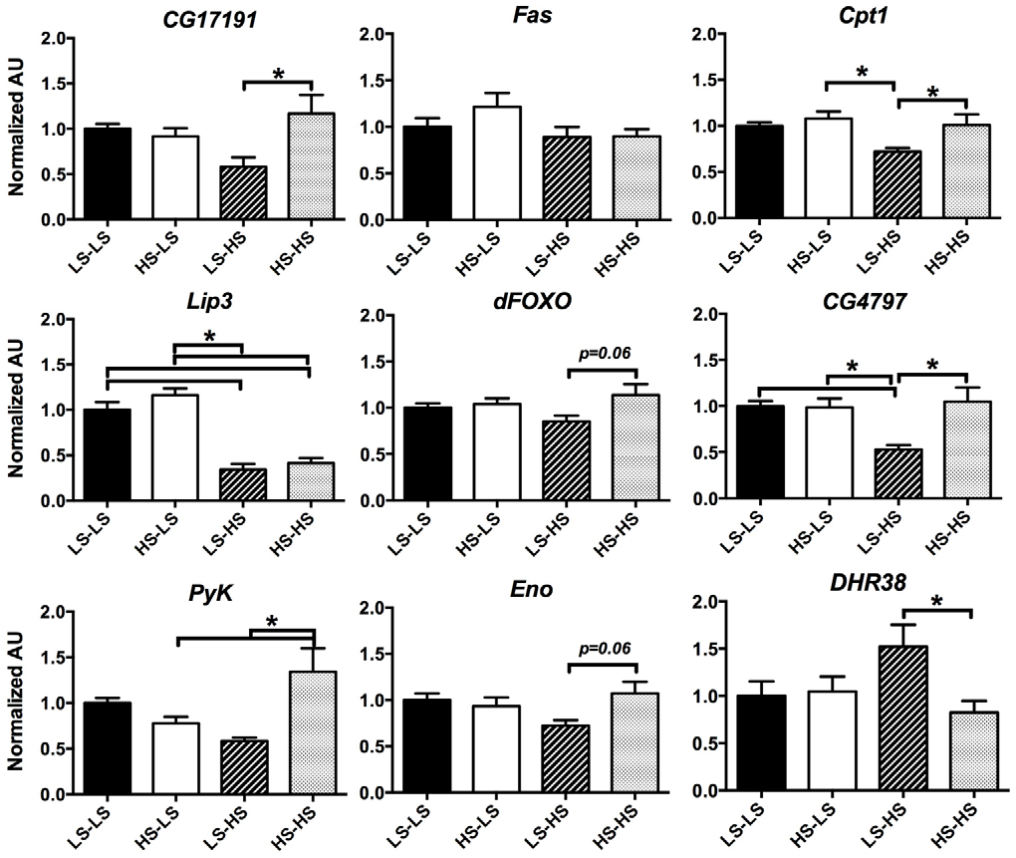
**Diet-challenged male adult offspring exhibit differential gene expression.** qRT-PCR analysis of mRNA from adult male offspring from either LS- or HS-fed maternal flies cultured on either LS (LS-LS, HS-LS) or HS (LS-HS, HS-HS) food for 14 days after eclosion. Transcripts of gene involved in lipid metabolism: *CG17191* (lipase), *Fas*, *Cpt1, Lip3*, *dFOXO*; and carbohydrate metabolism: *CG4797* (glucose transporter), *PyK*, *Eno* and *DHR38*. Bars represent mean relative expression ± s.e.m., presented as fold change versus the control value (=1.0) in each case. All expression data was normalized to α-tubulin 84B mRNA. *n*=10–14 pooled samples; *P*<0.05 versus *LS-LS.

Next, we examined gene expression in control and diet-challenged male offspring. Adult male offspring reared as larvae on LS food were cultured on either LS or HS food for 14 days. We then extracted RNA from the whole animals and evaluated similar gene targets as those evaluated at the mL3 stage. Of the targets we examined, adult offspring from HS-fed maternal flies did not exhibit any significant changes in gene expression as compared with control animals under LS culture conditions (Fig. 5). However, when adult offspring from LS- or HS-fed maternal flies were subjected to an HS diet, differential gene expression became apparent. When compared with LS-LS controls, offspring from LS-fed maternal flies responded to an HS diet (LS-HS) with a trend towards the downregulation of lipase *CG17191* and *CPT1*. In contrast, offspring from HS-fed maternal flies cultured on HS food (HS-HS) did not exhibit any change in *CG17191* and *CPT1* expression levels compared with LS-LS. Thus, when compared with LS-HS flies, HS-HS offspring demonstrated a significant difference in expression of *CG17191* and *CPT1* (Fig. 5). We also noted a trend towards differential expression of *dFOXO*: the HS-HS offspring had higher expression levels than LS-HS offspring (*P*=0.06). Several differential changes were also noted in carbohydrate metabolic gene targets. HS-HS offspring upregulated *PyK*, which was slightly decreased in LS-HS flies, and *CG4797* was downregulated in LS-HS offspring, but remained similar to controls in the HS-HS flies. Finally, *CG1864* (*DHR38*), a transcriptional regulator thought to be involved in carbohydrate homeostasis in *Drosophila* (Ruaud et al., 2011), trended towards increased expression in LS-HS offspring, but was significantly decreased in expression in HS-HS offspring when compared with LS-HS flies. Overall, these data demonstrate that the alterations in body composition in both larval offspring and diet-challenged adult offspring of HS-fed flies are accompanied by transcriptional changes of metabolic regulators when compared with offspring from LS-fed flies. Importantly, the gene expression changes in adult HS-HS offspring demonstrate a different gene regulatory response to caloric excess than in LS-HS flies, indicating an alternative metabolic programming mechanism associated with maternal diet.

### F_2_ larval offspring from HS-fed maternal flies exhibit altered body composition

**Fig. 6.**
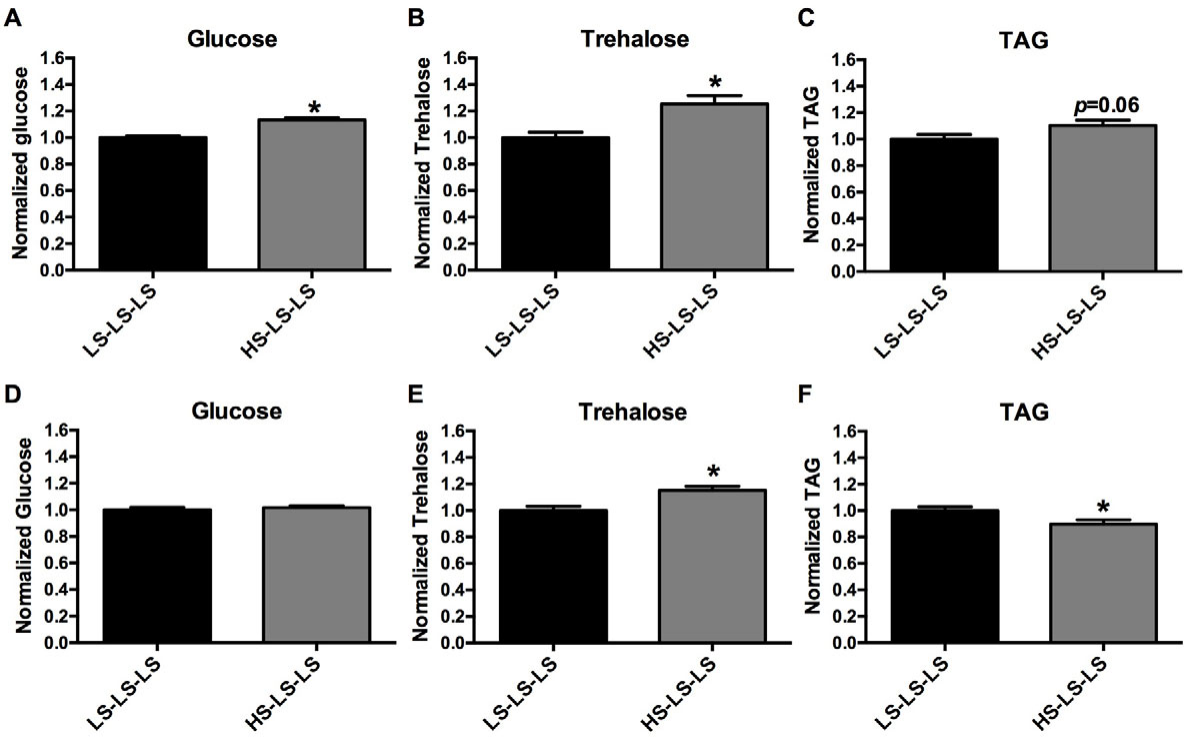
**Second-generation larval offspring have significant changes in body composition after a maternal HS diet.** wL3 total body composition of F_2_ generation male offspring (A–C) [(A) glucose, (B) trehalose, (C) TAG] and females (D–F) [(D) glucose, (E) trehalose, (F) TAG]. *x*-axis labels denote maternal diet-F_1_ larval and adult diets-F_2_ larval diet. Bars represent mean body composition, normalized to weight and presented as compared with control (LS-LS-LS=1.0) ± s.e.m.; *n*=20–30 pooled samples; *P<0.05 versus LS-LS-LS.

To determine whether subsequent generations of animals were affected by a maternal HS diet, we examined larval body composition of the F_2_ generation. Virgin females (F_0_) cultured on an LS or HS diet for 7 days were mated (as described above) to generate the F_1_ population. F_1_ virgin females were then cultured on LS food for 7 days prior to mating to generate the F_2_ population, which were also reared on the LS diet. Hence, only the F_0_ maternal flies were exposed to different diets (LS or HS), and the F_2_ offspring are denoted as either LS-LS-LS or HS-LS-LS (for F_0_ diet, F_1_ diet and F_2_ diet). Examination of body composition of wL3 male offspring from the F_2_ generation showed an increase in glucose and trehalose, similar to what was observed in the F_1_ generation (Fig. 6A,B). Additionally, TAG was slightly increased in these animals (Fig. 6C). Female wL3 offspring of the F_2_ generation were also examined. Although glucose was not altered in female offspring, trehalose was significantly increased (Fig. 6D,E). In contrast to F_2_ male offspring, F_2_ female offspring exhibited significantly decreased TAG (Fig. 6F). Therefore, alterations in body composition as a result of maternal HS diet are retained in the F_2_ generation. These data are strongly indicative of metabolic programming.

## DISCUSSION

**Fig. 7.**
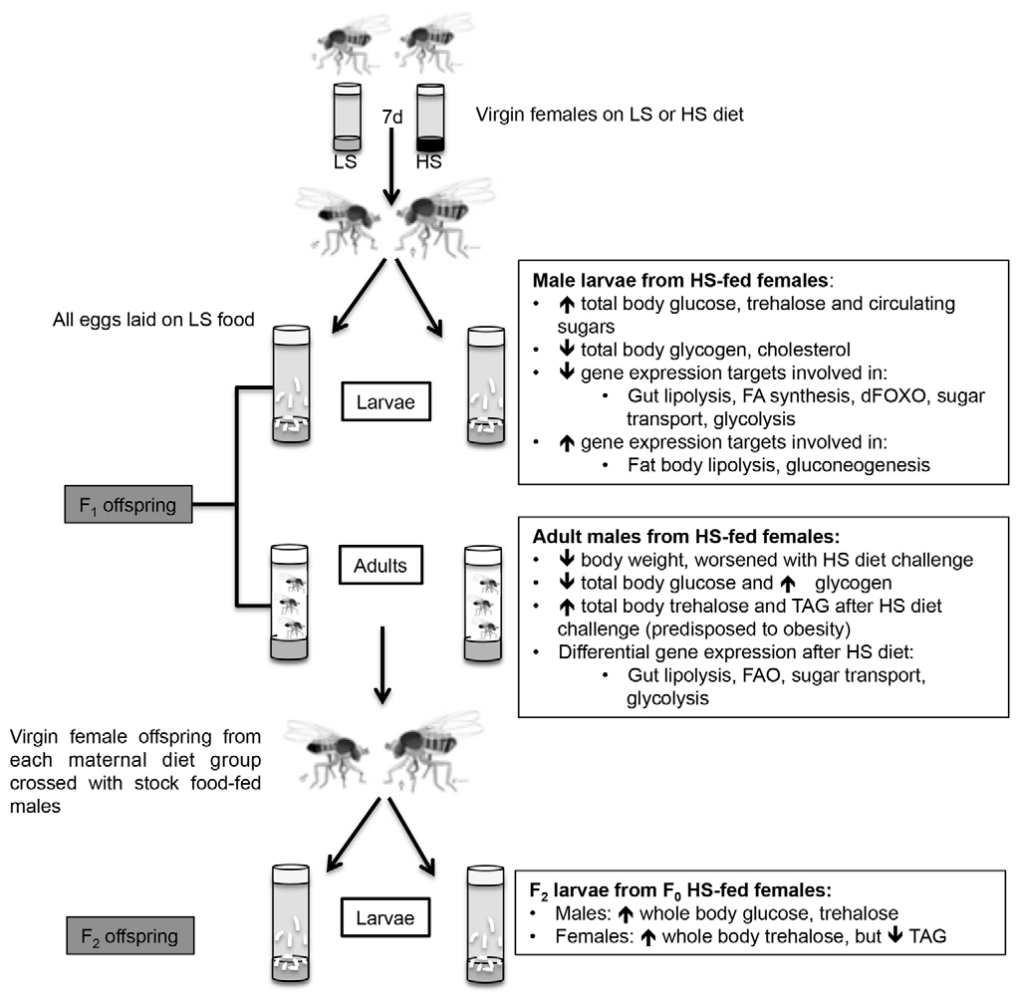
**Summary of experimental design and results.** Virgin female flies were placed on either an LS or HS diet for 7 days and then crossed with wild-type male flies (from stock food). All offspring developed on LS food. F_1_ offspring were examined at both the larval and adult stage and had altered body composition and expression of metabolic target genes. Virgin female F_1_ offspring were collected and crossed with stock-food-fed males. Both male and female F_2_ larval offspring had body composition changes. FAO, fatty acid oxidation.

We have established a new model for evaluating the impact of maternal caloric excess on offspring metabolism. We observed that obese-like maternal flies, generated by an HS feeding regimen, give rise to progeny with metabolic defects characterized by altered body composition and misregulation of metabolic genes (Fig. 7). Specifically, we found that wL3 offspring exhibited increased whole body and hemolymph glucose and trehalose, and modest reductions in glycogen and cholesterol. Increased whole body glucose and trehalose persisted in the F_2_ generation offspring. Despite the absence of TAG defects in the F_1_ wL3 population, F_2_ wL3 offspring also demonstrated changes in TAG composition. Furthermore, in addition to exhibiting increased glycogen storage under LS culture conditions, adult male offspring from HS-fed maternal flies seem to be predisposed to adiposity when challenged with an HS diet. The changes in both larval and adult offspring composition were also accompanied by altered gene expression of metabolic regulators. Taken together, this work supports the use of *Drosophila* as a novel model for examining the transgenerational effects of nutrition and lays the groundwork for future investigations into the molecular mechanisms underlying metabolic programming.

The developing larval offspring from HS-fed female flies have a mild phenotype that is primarily associated with altered carbohydrate homeostasis. The significant elevation in circulating sugar levels suggests an inability to take up and store carbohydrate. The gene expression changes in the larval offspring also demonstrate significant differences in the metabolic gene regulatory program that could be responsible for the altered body composition changes we found. Despite having elevated circulating sugar levels, we noted a mild decrease in whole body glycogen in wL3 animals. Interestingly, we observed a trend towards increased glucose-6-phosphatase expression, which would potentially drive more free glucose production; this seems counterproductive given the presence of hyperglycemia and hypertrehalosemia. Although we did not detect any significant changes in whole animal TAG levels in wL3 offspring, we did note changes in lipid metabolic gene expression. In particular, we documented opposing responses for two different lipases, *CG17191* and *CG8823. CG17191* is thought to be in the gastric lipase family (Horne et al., 2009), and is predominantly midgut expressed, which would suggest that it might play a role in gut lipid breakdown and absorption of free fatty acids. *CG8823* is most similar to mammalian lysosomal lipases (Pistillo et al., 1998), which are known to act on TAG and cholesterol esters. It is possible that some of these changes in gene expression are indirect or compensatory, as a result of feeding differences or the changes in body composition. For instance, the decrease in *CG17191* could represent an effort to decrease intestinal absorption of free fatty acids as a result of the existing excess of total body carbohydrates and circulating sugars, which might already be sustaining energy needs. The upregulation of *CG8823* would likely produce a decrease in total body TAG; it might also be differentially regulated in an effort to balance the excess of nutrient availability. We also noted decreased expression of two genes that normally drive fatty acid synthesis (*Fas* and *dACC*). This is interesting because it is probably beneficial for excess circulating sugars to be shuttled into TAG storage rather than remain circulating (Tan and Vidal-Puig, 2008). Thus, the combination of body composition and gene regulatory changes suggests that the overall balance between nutrient storage and catabolism is misregulated.

There is limited data in other animal models regarding the impact of maternal obesity or diabetes at early developmental stages. One study of maternal fructose intake in pregnant rats was associated with increased circulating glucose and fructose levels in young female offspring (Vickers et al., 2011). There was also an association between maternal fructose intake and altered leptin levels in offspring, suggesting possible neuroendocrine consequences of a maternal high-fructose diet. Additional evidence in mice indicates that exposure to trans-fatty acids during pregnancy and lactation alters glucose homeostasis and insulin signaling in neonatal pups (Kavanagh et al., 2010). In humans, there is abundant data that maternal adiposity is associated with offspring obesity (Boerschmann et al., 2010; Fraser et al., 2010; Hochner et al., 2012), but early developmental changes are unexplored. Our *Drosophila* model allows for probing the metabolic changes at multiple points in development, and consequently presents an opportunity for the discovery of mechanisms that alter early developmental metabolic pathways, providing a foundation for future focused studies in mammalian systems. Future studies could focus on insulin signaling as well as TOR pathway signaling, both of which have been implicated in high-calorie feeding models in flies (Birse et al., 2010).

Adult offspring from HS-fed maternal flies cultured on LS food throughout their lifespan exhibited trehalose and TAG composition similar to control animals. Yet, when transferred to HS food after eclosion, the accumulation of trehalose and TAG surpassed that of control animals fed an HS diet. Thus, offspring from HS-fed maternal flies demonstrate a predisposition to developing an obese-like phenotype during suboptimal, HS nutritional conditions. The predisposition to increased adiposity might contribute to metabolic disease; therefore, this diet challenge paradigm will serve as an important platform for studying underlying mechanisms that can contribute to ongoing disease states. Recent data in a mouse model of high-fat feeding linked prenatal combined with postnatal fat exposure to a worse phenotype, including obesity and cardiac hypertrophy, probably secondary to lipotoxicity, glucose intolerance and mitochondrial deficits (Turdi et al., 2013). Accordingly, our findings in the *Drosophila* model are similar to phenotypes seen in mammalian systems. Interestingly, the most robust increases in trehalose were observed in adult HS-fed females and their adult offspring after diet challenge, whereas larval changes were modest. The overall metabolic goals and demands are highly different in the adult stage compared with the larval stage. Indeed, *Drosophila* larvae are predominantly programmed for energy storage to ensure complete pupation and successful eclosion. Conversely, adult flies have greater energy demands because of flight and mating. The differences between larval and adult sugar levels might be related to these changing metabolic demands, and might suggest a preference or program in adults exposed to an HS diet to increase the synthesis of trehalose even after the glucose challenge has been removed. The predisposition to hyperglycemia and adiposity in offspring from HS-fed maternal flies was also accompanied by differential gene expression of enzymes that function in sugar and lipid metabolic pathways. This raises the possibility that early developmental adaptations to the maternal diet might program metabolic pathways in later life stages.

Under basal LS culture conditions, adult offspring from HS maternal flies exhibited increased glycogen stores, which further increased when these animals were cultured on HS food. Interestingly, increased glycogen correlated with a decrease in expression of *DHR38*, a nuclear receptor transcription factor. *DHR38* is a non-ligand-activated transcription factor in the NR4 family of nuclear receptors that has been implicated in carbohydrate homeostasis in *Drosophila* larvae (Ruaud et al., 2011). The study by Ruaud et al. showed that *DHR38* is necessary for glycogen synthesis and storage, and provided evidence that decreased *DHR38* activity was associated with decreased glycogen stores in larval offspring of control-diet-fed mothers (Ruaud et al., 2011). Therefore, *DHR38* might function in a diet-specific manner. It is important to note that the Ruaud study was in larvae, because mutant flies died at eclosion, and thus the role of *DHR38* in adult flies is not known. We evaluated DHR38 activity in offspring of LS- and HS-fed females using a GAL4-DHR38 fusion protein and were unable to detect any differences in DHR38 activity (data not shown). However, it is possible that the difference in activity is not robust enough to detect using this technique. Further studies will evaluate the role of *DHR38* in the adult offspring phenotype after a maternal HS diet using *DHR38* mutant lines.

Finally, we documented that body composition changes associated with a maternal HS diet persisted into a second generation of animals. Importantly, the change in the wL3 F_2_ larvae also included a mild increase in total body TAG. These data suggest that a predisposition to obesity can be passed on through multiple generations. There is some data in other animal models to support multigenerational effects of maternal obesity. Li et al. demonstrated that high-fat feeding for three consecutive generations resulted in increasing degrees of obesity across the generations (Li et al., 2012). Waterland et al. also noted successive worsening of obesity across generations of Agouti mice, and linked this predisposition to obesity with epigenetic changes from DNA methylation (Waterland et al., 2008). Importantly, this latter study emphasizes the potential for epigenetic mechanisms as a means of transgenerational metabolic programming. *Drosophila*, like mammals, are subject to chromatin state modification by both DNA methylation (Krauss and Reuter, 2011) and histone modification (Ringrose et al., 2004; Tzeng et al., 2007). DNA methylation in particular has been implicated in metabolic programming of selected genes in mammals (Gemma et al., 2009; Pinney and Simmons, 2012), whereas the role of histone methylation and acetylation has had limited exploration. Conveniently, histone modifications and the necessary ‘machinery’ for these processes has been very well studied in *Drosophila*, with much of the original evidence stemming from studies in this organism. The conservation of epigenetic mechanisms between flies and mammals provides further evidence for the utility of this *Drosophila* model as a discovery tool; our model allows for the rapid evaluation of programming across multiple generations of offspring and a broader platform for discovery, secondary to the wide range of genetic ‘tools’ available in flies.

Our data support the use of *Drosophila* as a novel model for examining the transgenerational effects of nutrition and lays the groundwork for future investigations into the molecular mechanisms underlying metabolic programming. Indeed, altered gene expression in offspring from HS-fed maternal flies together with altered body composition in the F_2_ generation is highly indicative of metabolic programming. By using genetically tractable *Drosophila* as a model of metabolic programming, we anticipate that investigation into the role of specific metabolic pathways and into the regulatory role of epigenetic alterations in multiple generations will lead to the rapid elucidation of programming mechanisms in a manner that is not feasible in higher organisms.

## MATERIALS AND METHODS

### Fly stocks

*w^1118^ Drosophila* stocks were obtained from the Bloomington *Drosophila* Stock Center and maintained at 25°C on molasses-based fly food. Virgin female flies were collected from stocks and placed within 24 hours of eclosion on either LS or HS food for 7 days (Fig. 7). The food content of LS and HS diets is listed in Table 1. Weights and whole body composition measurements were collected in groups of six larvae and groups of eight flies.

**Table 1.**
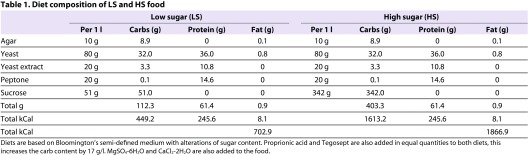
Diet composition of LS and HS food

### Whole body composition measurements

In order to appropriately stage larvae, preliminary studies were performed using food containing 0.05% bromophenol blue dye. This non-toxic dye is visible in the gut of the larva, and larvae that have truly stopped feeding lack this dye in the anterior portion of the midgut (Andres and Thummel, 1994). This allowed us to determine that animals in the upper half of the vial were true wanderers, because blue-gut larvae did not venture this far from the food. Whole body glucose, trehalose and glycogen content were determined by homogenizing pooled groups of six wL3 or eight adult animals in PBS or PBS + 0.1% Tween. Values were normalized to group weight then to LS values (LS=1). For glucose measurements, 2 μl of homogenate was combined with 98 μl of Infinity™ Glucose Hexokinase Liquid Stable Reagent (Fisher) and incubated at 37°C for 15 minutes. Trehalose was measured as described (Musselman et al., 2011) using 5 μl of homogenate. For glycogen, homogenates were centrifuged briefly at 2400 ***g***, supernatant was transferred, centrifuged at 18,800 ***g*** and then heated at 70°C for 5 minutes. Larval homogenates were diluted 1:3 and fly homogenates were diluted 1:7.5, and glycogen was measured as described (Palanker et al., 2009). Whole body TAG content was determined by homogenizing animals in PBS + 0.1% Tween as described (Musselman et al., 2011). Absorbance was measured at 340 nm or 560 nm with a microplate reader (Tecan Infinite^®^ 200 PRO). Whole body cholesterol content was determined as previously reported (Horner et al., 2009) using the Amplex^®^ Red Cholesterol Assay Kit (Invitrogen). Relative fluorescent units (RFU) were determined using a Tecan Infinite 200^®^ PRO microplate reader.

### Hemolymph glucose and trehalose measurements

Hemolymph was pooled from ten larvae to obtain ∼1 μl for assays. Hemolymph glucose and trehalose were quantified as previously reported (Musselman et al., 2011).

### qRT-PCR

mL3 animals were staged by selecting animals that had undergone the L2/L3 molt and then aging them an additional 24 hours. Thus, all animals were at the mid-point of the third instar stage. RNA was extracted from pooled samples of 15 mL3 larvae and adult flies using TRIzol^®^ (Invitrogen), and was then DNase treated (NEB) and purified on RNeasy columns (Qiagen). RNA was then subjected to reverse transcription followed by qRT-PCR using SYBR^®^Green (Applied Biosystems). Reactions were performed in triplicate. SYBR^®^Green intercalation was measured using an Mx3005P (Stratagene). Target mRNA expression levels were normalized to levels of α-tubulin 84B mRNA. Relative quantification after normalization was calculated using the 2^−ΔCT^ formula. Primer sequences for qRT-PCR are available upon request.

### Statistics

Each reported replicate represents a pooled sample of animals that were selected from different crosses within a single experiment. All data includes at least three experimental replicates from different days. All data are expressed as mean ± standard error of the mean (s.e.m.). Comparisons between data were done by unpaired, two-tailed Student’s *t*-test or one-way analysis of variance (ANOVA) coupled with Tukey’s test for multiple comparisons.
